# Impact of anesthesia methods on functional outcomes in patients with acute ischemic stroke undergoing mechanical thrombectomy: insights from the ENCHANTED2/MT trial

**DOI:** 10.3389/fneur.2026.1736293

**Published:** 2026-04-23

**Authors:** Hao Wang, Hongye Xu, Xiongfeng Wu, Xiaoxi Zhang, Rundong Chen, Lei Zhang, Xiaofei Ye, Yongwei Zhang, Pengfei Yang, Shengdong Chen, Xin You, Jianmin Liu

**Affiliations:** 1 Department of Neurology, Linyi People's Hospital, Affiliated Hospital of Shandong Second Medical University, Linyi, China; 2 Neurovascular Center, Changhai Hospital, Naval Medical University, Shanghai, China; 3 No. 904 Hospital of the PLA Joint Logistics Support Force, Wuxi, China; 4 Changhai Clinical Research Unit, Naval Medical University, Shanghai, China; 5 Department of Statistics, Naval Medical University, Shanghai, China; 6 Department of Health Management Center, Linyi People's Hospital, Affiliated Hospital of Shandong Second Medical University, Shandong, China

**Keywords:** acute ischaemic stroke, endovascular thrombectomy, anesthesia modality, functional independence, randomized trial

## Abstract

**Background:**

The choice of anesthesia modality during mechanical thrombectomy (MT) for acute ischemic stroke remains debated. This study aimed to analyze the impact of anesthesia type on clinical outcomes following endovascular therapy (EVT).

**Methods:**

This subgroup analysis of the ENCHANTED2/MT trial included patients with large vessel occlusion (LVO) acute ischemic stroke who achieved successful reperfusion (eTICI ≥2b). Patients were stratified by anesthesia type: general anesthesia (GA) or non-general anesthesia (Non-GA). The primary outcome was functional recovery, assessed by the distribution of scores on the modified Rankin Scale (mRS; range 0 [no symptoms] to 6 [death]) at 90 days. Efficacy was analyzed using ordinal logistic regression.

**Results:**

A total of 809 patients were included (GA: 344; Non-GA: 465). There was no significant between-group difference in the primary outcome of mRS score distribution. The incidence of postoperative pneumonia was significantly lower in the Non-GA group (adjusted odds ratio [aOR] 0.33, 95% CI 0.14–0.74; *p* = 0.008). Among patients with milder initial neurological deficits (admission NIHSS score <15), the Non-GA group had higher odds of achieving functional independence (mRS 0–2) at 90 days (aOR 1.71, 95% CI 1.00–2.91; *p* = 0.049); this association was not observed in patients with more severe deficits (NIHSS ≥15) (P for interaction = 0.05). No significant differences were found between groups in the rates of symptomatic intracranial haemorrhage, recurrent stroke, mortality, or in the level of reperfusion.

**Conclusion:**

In patients undergoing mechanical thrombectomy for acute ischemic stroke, Non-GA may be associated with better functional outcomes in patients presenting with milder neurological deficits. GA was associated with an increased risk of postoperative pneumonia.

## 1 Introduction

Acute ischemic stroke (AIS) due to large vessel occlusion (LVO) represents a major global cause of mortality and long-term disability (1). The advent of mechanical thrombectomy (MT) has revolutionized treatment, offering the potential for dramatic functional recovery by achieving rapid cerebral reperfusion. Its efficacy is firmly established, and it is now the standard of care for eligible patients as per international guidelines (2–4). The procedural success of MT, however, is contingent not only on technical recanalization but also on meticulous peri-procedural management. Among the most debated aspects of this management is the choice of anesthetic modality: general anesthesia (GA) versus non-general anesthesia (Non-GA, encompassing conscious sedation or local anesthesia alone). Proponents of GA argue that it guarantees patient immobility, optimizes imaging conditions, secures the airway, and allows for controlled ventilation and hemodynamics, conversely, advocates for Non-GA highlight the potential avoidance of GA-induced hypotension, the preservation of cerebral autoregulation, and the reduction in procedure-related delays and systemic complications (5–9).

The existing body of evidence presents a complex and often contradictory picture. Early observational studies and meta-analyses frequently associated GA with worse neurological outcomes. In contrast, several randomized controlled trials (RCTs), such as SIESTA, GOLIATH, and ANSTROKE, reported no significant difference in primary functional outcomes between the two strategies. Recent multicenter RCTs like AMETIS and SEGA have further added to the inconsistency, reporting neutral and positive effects for GA, respectively. This heterogeneity likely stems from differences in study design, patient populations, and crucially, from significant confounding by indication in non-randomized comparisons, where sicker patients are more likely to receive GA (10, 11).

The ENCHANTED2/MT trial, a pivotal multicenter randomized controlled trial conducted across 44 tertiary hospitals in China, compared intensive versus standard blood-pressure management following successful mechanical thrombectomy. The primary results demonstrated that intensive blood-pressure lowering (target systolic blood pressure <120 mmHg) after successful recanalization may lead to poorer functional outcomes in patients with acute large-vessel occlusion stroke (12). To provide further evidence from an East Asian population, this prespecified subgroup analysis of the trial was performed to evaluate the impact of different anesthesia modalities on both early and late functional outcomes.

## 2 Methods

### 2.1 Study design and participants

This was a *post hoc* subgroup analysis of data from the ENCHANTED2/MT trial—a multicentre, randomized, controlled trial that compared intensive (target systolic blood pressure [SBP] < 120 mmHg) versus standard (target SBP 140–180 mmHg) blood pressure management following successful endovascular thrombectomy for acute ischemic stroke due to large vessel occlusion. The full trial design, inclusion and exclusion criteria, treatment protocol, and primary results have been published previously (12, 13). For the present analysis, we included all trial participants for whom anesthesia strategy data were available. Patients were stratified according to the anesthetic approach used during the procedure: general anesthesia (GA) or non-general anesthesia (Non-GA; encompassing local anesthesia alone or conscious sedation). The choice of anesthesia modality was at the discretion of the treating team at each participating center. The trial protocol received approval from the ethics committee or institutional review board at every participating site, and written informed consent was obtained from all patients or their legally authorised representatives.

### 2.2 Outcomes

The primary outcome was functional recovery, evaluated as a shift in the distribution of scores on the modified Rankin Scale (mRS) at 90 days. The mRS is a standardised 7-level global disability scale, where a score of 0 indicates no symptoms, 1 indicates no clinically significant disability despite symptoms, 2–5 indicate increasing levels of disability and dependency, and 6 indicates death. Secondary outcomes included dichotomised assessments of the mRS at 90 days: excellent recovery (mRS 0–1), functional independence (mRS 0–2), and moderate recovery (mRS 0–3). Other secondary efficacy and safety outcomes were: neurological impairment at day 7, defined as a worsening shift across seven categories of the National Institutes of Health Stroke Scale (NIHSS) score (<5, 5–9, 10–14, 15–19, 20–24, and ≥25); symptomatic intracranial haemorrhage (sICH), adjudicated primarily according to the Heidelberg Bleeding Classification criteria (14) and secondarily by the criteria of the NHImplementation of Thrombolysis in Stroke-Monitoring Study; all-cause mortality within 90 days; pneumonia, defined as a pulmonary infection detected by CT scan within one week after thrombectomy, was identified and reported by each local center and then centrally adjudicated and recorded; level of reperfusion, defined by the first angiographically assessed expanded Thrombolysis in Cerebral Infarction (eTICI) score (2b, 2c, or 3, on a scale from 0 [no perfusion] to 3 [complete perfusion]); and recurrent ischemic stroke, as determined by an independent clinical events committee blinded to treatment allocation, based on clinical presentation with a distinct symptom profile, imaging evidence of a new ischemic territory, angiographic confirmation of re-occlusion, or occurrence after a period of clinical stability following the index event.

### 2.3 Statistical analysis

Data are presented as median with interquartile range (IQR) for continuous variables and as percentages for categorical variables. Distribution normality was assessed using histograms and the Shapiro–Wilk test. Baseline characteristics were compared between the GA and Non-GA groups using the *χ*^2^ test or Cochran–Mantel–Haenszel test (for stratified categorical data) for categorical variables, and the Wilcoxon rank-sum test for continuous variables with non-Gaussian distributions. After verifying the proportional odds assumption using the Brant test, the association between anesthesia modality and the ordinal primary outcome (mRS at 90 days) was analysed using ordinal logistic regression, yielding a common odds ratio (OR). To account for potential between-center practice differences and the correlation of patient outcomes within centers, we performed a sensitivity analysis using study center as the clustering variable. Cluster-robust standard errors were applied to correct the standard errors of the primary ordinal logistic regression model. Specifically, the *vcovCL* function from the R *sandwich* package was used to compute the cluster-robust variance–covariance matrix with center as the clustering variable, and the *coeftest* function from the *lmtest* package was used to obtain the adjusted *p*-values and confidence intervals. Multivariable binary logistic regression was used to assess the relationship between anesthesia type and each dichotomous secondary outcome. Results are reported as unadjusted and adjusted ORs with corresponding 95% confidence intervals (CIs). The adjustment model included the following pre-specified covariates: age, sex, medical history of hypertension, previous stroke, coronary artery disease, use of antihypertensive medication, use of aspirin or other antiplatelet drugs, anticoagulation therapy, admission NIHSS score, admission Glasgow Coma Scale (GCS) score, time from triage to reperfusion, presence of early ischemic changes on baseline CT scan, use of intravenous alteplase, use of heparin, and the determined cause of large-vessel occlusion. A subgroup analysis for the outcome of functional independence (mRS 0–2) at 90 days was performed using multivariable logistic regression adjusted for covariates. Interaction effects were tested for the following dichotomised subgroups: age (<70 vs. ≥70 years); admission stroke severity (NIHSS score <15 vs. ≥15); time from symptom onset to triage (<6 h vs. ≥6 h); presence of early ischemic changes on baseline CT scan (yes vs. no); intensity of assigned blood pressure management strategy (intensive vs. standard); and use of intravenous alteplase prior to thrombectomy (yes vs. no). The common odds ratio represents the odds of worse outcomes for the Non-GA anesthesia group compared with the GA group. All analyses were performed using SAS (version 9.4) and R (version 4.1.3) software. A two-sided *p* value <0.05 was considered statistically significant.

## 3 Result

### 3.1 Study population and baseline characteristics

**Figure 1:**
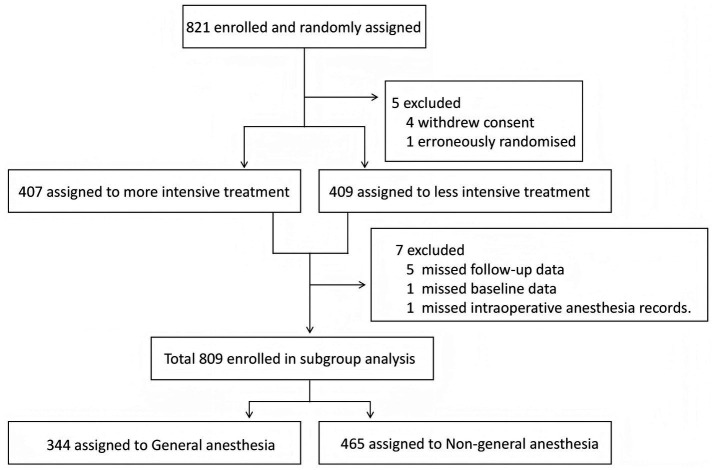
The flowchart of study population in this study.

The patient selection flowchart is presented in Figure 1. Within the ENCHANTED2/MT trial (12), 821 patients were enrolled between July 20, 2020, and March 7, 2022. Of these, we excluded one patient who did not meet the inclusion criteria, four who withdrew consent, five with missing follow-up data, one with missing baseline data, and one with missing intraoperative anesthesia records. Consequently, 809 patients were included in the final analysis. GA and Non-GA were administered to 344 and 465 patients, respectively.

The baseline demographic and clinical characteristics of the patients are summarized in Table 1. The median age was identical in both groups at 68 years. Males comprised 63.85% of the GA group and 60.3% of the Non-GA group. Compared to the GA group, patients in the Non-GA group had a lower prevalence of hypertension (60.52% vs. 69.97%, *p* = 0.005), coronary artery disease (10.73% vs. 16.91%, *p* = 0.011), and a trend toward a lower prevalence of previous stroke (8.58% vs. 12.83%, *p* = 0.05). Pre-admission use of antihypertensive drugs (34.76% vs. 54.81%, *p* < 0.001) and aspirin or other antiplatelet drugs (7.08% vs. 11.37%, *p* = 0.034) was also less common in the Non-GA group, while the use of anticoagulation drugs was more common (6.22% vs. 2.92%, *p* = 0.03). Regarding stroke severity and procedural metrics, patients in the Non-GA group presented with lower median admission NIHSS scores (14 vs. 16, *p* = 0.001), higher median Glasgow Coma Scale (GCS) scores (12 vs. 11, *p* < 0.001), and had a significantly shorter median time from triage to reperfusion (0.82 h vs. 1.33 h, *p* < 0.001). Signs of cerebral ischemia on the baseline CT scan were observed less frequently in the Non-GA group (27.74% vs. 51.36%, *p* < 0.001). The use of intravenous alteplase (27.25% vs. 34.4%, *p* = 0.029) and intravenous heparin (51.07% vs. 61.52%, *p* = 0.003) was lower in the Non-GA group. The determined etiology of the large-vessel occlusion also differed significantly between the two groups (*p* = 0.007).

**Table 1:** Patient characteristics at baseline and after endovascular thrombectomy treatment.

Characteristics	General anesthesia (*n* = 344)	Non-general anesthesia (*n* = 465)	*p* value
Age, y, median (IQR)	68 (59, 75)	68 (59.25, 76)	0.403
Male, *n* (%)	219 (63.85%)	281 (60.3%)	0.305
Hypertension, *n* (%)	240 (69.97%)	282 (60.52%)	0.005
Diabetes, *n* (%)	77 (22.45%)	84 (18.03%)	0.119
Coronary artery disease, *n* (%)	58 (16.91%)	50 (10.73%)	0.011
Valvular heart disease, *n* (%)	9 (2.62%)	23 (4.94%)	0.095
Other heart disease, *n* (%)	12 (3.5%)	22 (4.72%)	0.392
Atrial fibrillation, *n* (%)	75 (21.87%)	105 (22.53%)	0.822
Hypercholesterolaemia, *n* (%)	14 (4.08%)	13 (2.79%)	0.312
Previous stroke, *n* (%)	44 (12.83%)	40 (8.58%)	0.05
Antihypertensive drug, *n* (%)	188 (54.81%)	162 (34.76%)	<0.001
Statin or other lipid-lowering drug, *n* (%)	28 (8.16%)	32 (6.87%)	0.487
Aspirin or other antiplatelet drug, *n* (%)	39 (11.37%)	33 (7.08%)	0.034
Anticoagulation drug, *n* (%)	10 (2.92%)	29 (6.22%)	0.03
Admission systolic blood pressure, mm Hg, median (IQR)	157 (144, 173.5)	156 (144, 172)	0.603
Admission diastolic blood pressure, mm Hg, median (IQR)	89 (78, 99.5)	89 (80, 98)	0.543
Admission NIHSS, median (IQR)^a^	16 (12, 22)	14 (10, 20)	0.001
Admission GCS score, median (IQR)^b^	11 (8, 14)	12 (9, 15)	<0.001
Time from symptom onset to reperfusion, h, median (IQR)	7.37 (5.42, 10.22)	7.38 (4.68, 12.36)	0.905
Time from symptom onset to triage, h, median (IQR)	6.05 (4.01, 8.63)	6.41 (3.67, 11.4)	0.169
Time from triage to reperfusion, h, median (IQR)	1.33 (0.82, 2)	0.82 (0.57, 1.21)	<0.001
Signs of cerebral ischaemia on CT scan, *n* (%)	170 (51.36%)	124 (27.74%)	<0.001
Signs of cerebral infarction on MRI, *n* (%)	58 (69.05%)	39 (68.42%)	0.937
Use of intravenous alteplase, *n* (%)	118 (34.4%)	127 (27.25%)	0.029
Use of heparin, *n* (%)	211 (61.52%)	238 (51.07%)	0.003
Use of tirofiban, *n* (%)	158 (46.06%)	209 (44.85%)	0.732
More intensive treatment, *n* (%)	172 (50.15%)	232 (49.79%)	0.919
Cause of large-vessel occlusion, *n* (%)			0.007
Intracranial atherosclerosis	181 (52.77%)	207 (44.42%)	
Extracranial atherosclerosis	12 (3.5%)	18 (3.86%)	
Cardioembolism from atrial fibrillation	102 (29.74%)	127 (27.25%)	
Cardioembolism from other source	13 (3.79%)	37 (7.94%)	
Dissection	6 (1.75%)	7 (1.5%)	
Uncertain	29 (8.45%)	70 (15.02%)	
24 h mean systolic blood pressure after procedure, mm Hg, median (IQR)	129 (119.17, 142.29)	127.75 (117.52, 141.31)	0.153
24 h mean diastolic blood pressure after procedure, mm Hg, median (IQR)	72.58 (66, 80.83)	72.54 (65.6, 79.5)	0.568

Data are median (IQR), or *n*/*N* (%). NIHSS=National Institutes of Health Stroke Scale. GCS = Glasgow coma scale. eTICI = expanded Treatment In Cerebral Infarction.

a Scores on the NIHSS range from 0 to 42, with higher scores indicating more severe neurological deficits.

b Scores on the GCS range from 15 (normal) to 3 (deep coma).

### 3.2 Clinical outcome

**Figure 2:**
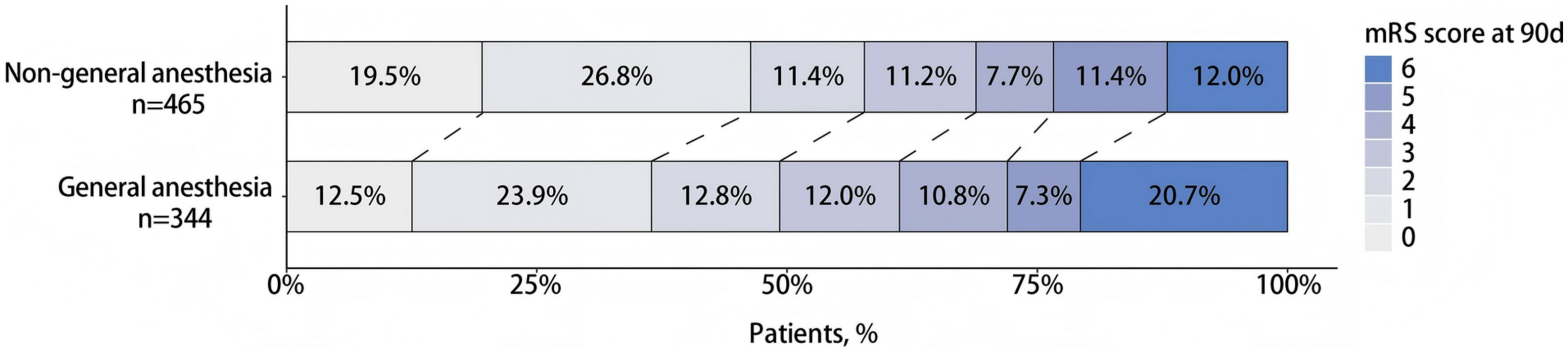
The distribution of modified Rankin Scale (mRS) at 90 days between general anesthesia and non-general anesthesia group.

Before adjustment, patients in the Non-GA group showed better functional outcomes on the modified Rankin Scale (mRS) compared to the GA group (unadjusted OR 0.66, 95% CI 0.52–0.85; *p* = 0.001), as detailed in Table 2 and illustrated in Figure 2. However, after adjusting for covariates, this difference was no longer statistically significant (adjusted OR 0.78, 95% CI 0.59–1.03; *p* = 0.077). Cluster-robust standard error analysis showed that the association between anesthesia modality and the primary outcome was consistent with the main analysis (adjusted OR 0.78, 95% CI 0.58–1.05, *p* = 0.094), indicating that center-level differences did not substantively alter the conclusions. The Brant test indicated no violation of the parallel regression assumption (*χ*^2^ = 8.18, df = 5, *p* = 0.15).

**Table 2:** Association between clinical outcomes and the different anesthesia methods.

Outcomes	General anesthesia	Non-general anesthesia	Unadjusted OR(95%CI)	*p* value	Adjusted^a^ OR(95%CI)	*p* value
Primary outcome						
Ordinal analysis of category scores on the mRS^b^			0.66 (0.52, 0.85)^c^	0.001	0.78 (0.59, 1.03)^c^	0.077
0 (no symptoms at all), *n* (%)	43 (12.54%)	91 (19.53%)				
1 (no significant disability despite symptoms), *n* (%)	82 (23.91%)	125 (26.82%)				
2 (slight disability), *n* (%)	44 (12.83%)	53 (11.37%)				
3 (moderate disability requiring some help), *n* (%)	41 (11.95%)	52 (11.16%)				
4 (moderate–severe disability requiring assistance with daily living), *n* (%)	37 (10.79%)	36 (7.73%)				
5 (severe disability, bed-bound, and incontinent), *n* (%)	25 (7.29%)	53 (11.37%)				
6 (death), *n* (%)	71 (20.7%)	56 (12.02%)				
Secondary outcomes						
mRS of 0–1 at 90 days, *n* (%)^j^	125 (36.44%)	216 (46.35%)	1.51 (1.13, 2)	0.005	1.37 (0.98, 1.92)	0.067
mRS of 0–2 at 90 days, *n* (%)^k^	169 (49.27%)	269 (57.73%)	1.41 (1.06, 1.86)	0.017	1.2 (0.85, 1.68)	0.305
mRS of 0–3 at 90 days, *n* (%)^l^	210 (61.22%)	321 (68.88%)	1.4 (1.05, 1.88)	0.024	1.18 (0.82, 1.7)	0.36
Ordinal analysis of category scores for neurological impairment at day 7			0.67 (0.52, 0.86)^d^	0.002	0.83 (0.62, 1.11)^d^	0.215
<5, *n* (%)	117 (34.11%)	197 (42.27%)				
5–9, *n* (%)	54 (15.74%)	90 (19.31%)				
10–14, *n* (%)	64 (18.66%)	63 (13.52%)				
15–19, *n* (%)	31 (9.04%)	39 (8.37%)				
20–24, *n* (%)	10 (2.92%)	22 (4.72%)				
≥25, *n* (%)	67 (19.53%)	55 (11.8%)				
Death at 90 days, *n* (%)	71 (20.7%)	56 (12.02%)	0.52 (0.36, 0.77)	0.001	0.69 (0.44, 1.07)	0.096
Symptomatic intracranial haemorrhage, *n* (%)^g^	28 (8.16%)	19 (4.08%)	0.48 (0.26, 0.87)	0.016	0.61 (0.31, 1.23)	0.169
Pneumonia, *n* (%)^h^	26 (7.58%)	10 (2.15%)	0.27 (0.13, 0.56)	0.001	0.33 (0.14, 0.74)	0.008
Recurrent ischaemic stroke, *n* (%)^i^	19 (5.54%)	26 (5.58%)	1.01 (0.55, 1.85)	0.98	1.14 (0.56, 2.32)	0.724
Ordinal analysis of category scores for eTICI score at the end of the procedure (level of reperfusion)			0.89 (0.62, 1.29)^f^	0.542	0.85 (0.56, 1.29)	0.43
2b, *n* (%)	30 (8.75%)	40 (8.58%)				
2c, *n* (%)	27 (7.87%)	46 (9.87%)				
3, *n* (%)	286 (83.38%)	380 (81.55%)				

mRS = modified Rankin scale. NIHSS=National Institutes of Health Stroke Scale. eTICI = expanded Treatment In Cerebral Infarction. The common odds ratio represents the odds of worse outcomes for the non-general anesthesia group compared with the general anesthesia group.

a Values adjusted for age, sex, medical history of hypertension, previous stroke and coronary artery disease, antihypertensive drugs, aspirin or other antiplatelet drug, anticoagulation drug, admission NIHSS, admission GCS score, time from triage to reperfusion, signs of cerebral ischaemia on CT scan, use of intravenous alteplase, use of heparin, cause of large-vessel occlusion.

b The mRS evaluates global disability; scores range from 0 (no symptoms) to 6 (death); a score of 2–5 indicates some degree of disability.

c Estimated from an ordinal logistic regression model and indicates the common odds of worse functional outcome for the non-general anesthesia group compared with the general anesthesia group.

d Estimated from an ordinal logistic regression model and indicates the odds of worse neurological deterioration measured on the NIHSS for the non-general anesthesia group compared with the general anesthesia group; scores on the NIHSS range from 0 to 42, with higher scores indicating more severe neurological deficits.

f Estimated from an ordinal logistic regression model and indicates the odds of worse reperfusion level defined as the first visualisation of successful reperfusion, as indicated by an eTICI score of 2b, 2c, or 3 (on a scale from 0 [no reperfusion] to 3 [complete reperfusion]).

g Symptomatic intracranial haemorrhage was defined as a haematoma occupying ≥30% of the infarcted tissue with obvious mass effect, as judged by an adverse-event committee as per Heidelberg criteria.

h Pneumonia was defined as a pulmonary infection detected by CT scan within one week after thrombectomy.

i Adjudicated by an adverse-event committee unaware of treatment allocation according to the definition of an ischaemic event with a different symptom profile, ischaemic location on the imaging report, recanalisation on angiography, or after a stable time period, from the index ischaemic stroke event.

j Excellent recovery at 90 days defined as an mRS score of 0 to 1.

k Functional independence at 90 days was defined as an mRS score of 0 to 2.

l Moderate recovery at 90 days defined as an mRS score of 0 to 3.

In unadjusted analyses of secondary outcomes, the Non-GA group exhibited higher rates of excellent recovery (mRS 0–1) at 90 days (unadjusted OR 1.51, 95% CI 1.13–2.00; *p* = 0.005), functional independence (mRS 0–2) at 90 days (unadjusted OR 1.41, 95% CI 1.06–1.86; *p* = 0.017), and moderate recovery (mRS 0–3) at 90 days (unadjusted OR 1.40, 95% CI 1.05–1.88; *p* = 0.024). Additionally, neurological impairment at day 7 was less severe in the Non-GA group (unadjusted OR 0.67, 95% CI 0.52–0.86; *p* = 0.002). The incidence of symptomatic intracranial haemorrhage (unadjusted OR 0.48, 95% CI 0.26–0.87; *p* = 0.016), 90-day mortality (unadjusted OR 0.52, 95% CI 0.36–0.77; *p* = 0.001), and pneumonia (unadjusted OR 0.27, 95% CI 0.13–0.56; *p* = 0.001) were all lower in the Non-GA group. No significant differences were observed between the groups in the rate of recurrent ischemic stroke or in the level of reperfusion. After adjustment for the following covariates—age, sex, medical history of hypertension, previous stroke, coronary artery disease, antihypertensive medication, use of aspirin or other antiplatelet drugs, anticoagulation therapy, admission NIHSS score, admission GCS score, time from triage to reperfusion, signs of cerebral ischemia on CT scan, use of intravenous alteplase, use of heparin, and cause of large-vessel occlusion—the Non-GA group maintained a significantly lower risk of postoperative pneumonia compared to the GA group (adjusted OR 0.33, 95% CI 0.14–0.74; *p* = 0.008). No other significant differences in clinical outcomes were observed following adjustment (Table 2).

**Figure 3:**
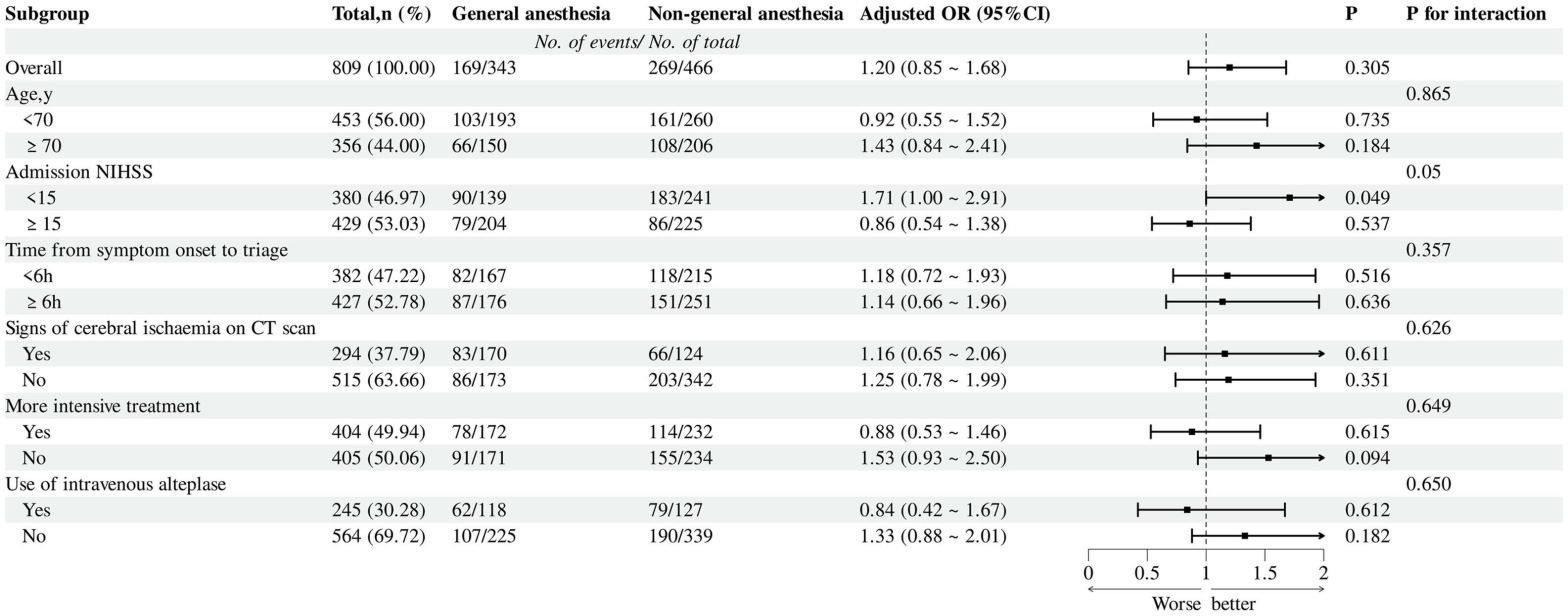
Subgroup analysis for the favorable functional outcome in each dichotomized subgroup.

**Figure 4:**
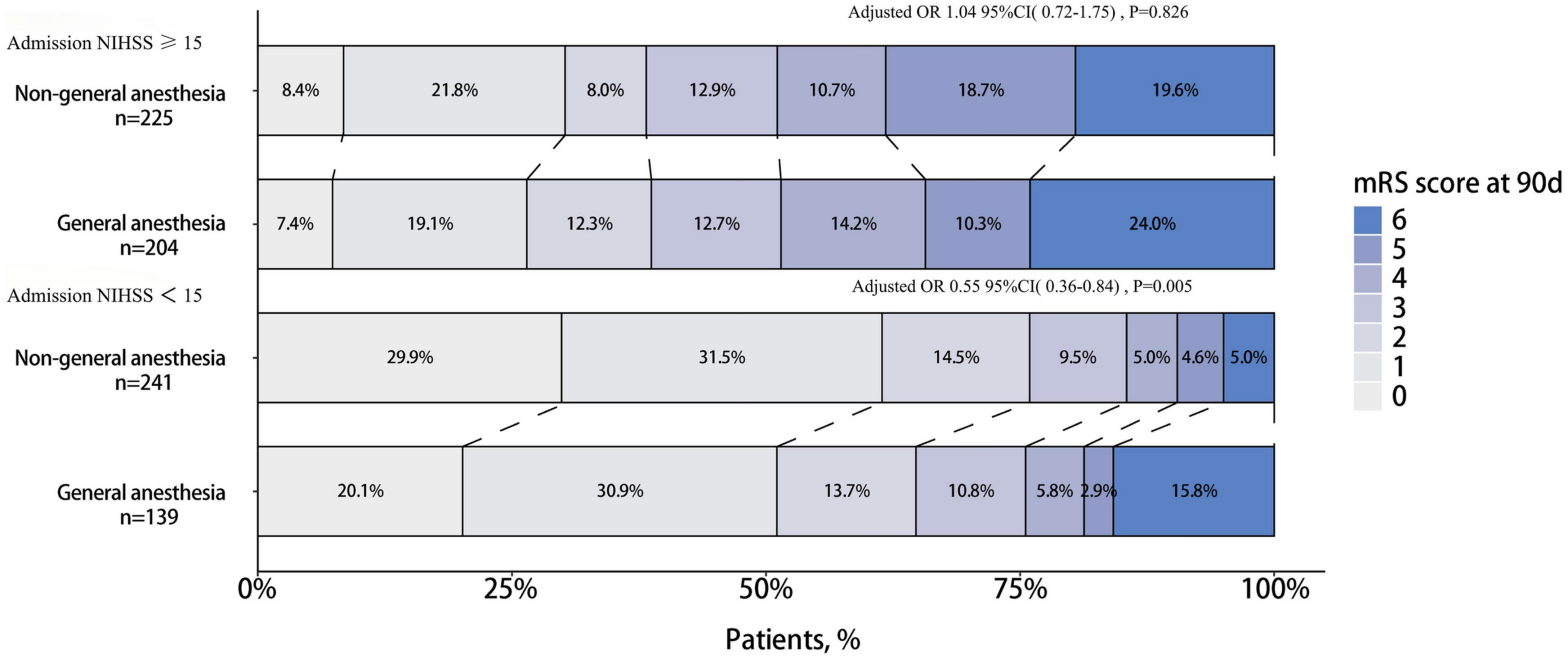
The distribution of modified Rankin Scale (mRS) at 90 days between general anesthesia (GA) and non-general anesthesia (Non-GA) group in two different admission NIHSS score ranges. After covariates adjustment using ordinal logistic regression, patients with an NIHSS score <15 in the Non-GA group demonstrated significantly lower mRS scores compared to the GA group (adjusted OR 0.55, 95% CI 0.36–0.84, *p* = 0.005). No significant between-group difference was observed in patients with an NIHSS score ≥15 (adjusted OR 1.04, 95% CI 0.72–1.75, *p* = 0.826).

In the subgroup analysis of functional independence (mRS 0–2) at 90 days, after covariate adjustment, patients with milder neurological deficits on admission (NIHSS <15) who received Non-GA were more likely to achieve functional independence than those in the GA group (adjusted OR 1.71, 95% CI 1.00–2.91; *p* = 0.049). This association was not observed in patients with more severe deficits on admission (NIHSS ≥15) (P for interaction = 0.05) (Figure 3). After covariate adjustment using ordinal logistic regression, patients with an NIHSS score <15 in the Non-GA group demonstrated significantly lower mRS scores compared to the GA group (adjusted OR 0.55, 95% CI 0.36–0.84, *p* = 0.005). No significant between-group difference was observed in patients with an NIHSS score ≥15 (adjusted OR 1.04, 95% CI 0.72–1.75, *p* = 0.826) (Figure 4). No other significant interactions were identified across the remaining binary subgroups.

## 4 Discussion

This investigation, anchored in the ENCHANTED2/MT trial, examines the influence of anesthetic modality on clinical outcomes for patients with acute ischemic stroke undergoing MT. After comprehensive multivariable adjustment, while the between-group difference in the primary outcome—the distribution of 90-day mRS scores—did not reach the conventional threshold for statistical significance (adjusted odds ratio [aOR] 0.78, 95% CI 0.59–1.03, *p* = 0.077). However, in exploratory subgroup analysis, we identified that for patients presenting with milder neurological impairment on admission (NIHSS score <15), undergoing the procedure under Non-GA was independently associated with a significantly higher likelihood of achieving functional independence (mRS 0–2) at 90 days (aOR 1.71, 95% CI 1.00–2.91, *p* = 0.049). This association was not evident in patients with moderate-to-severe deficits (NIHSS score ≥15; P for interaction = 0.05). Furthermore, this study suggests an association between GA and an increased risk of postoperative pneumonia (aOR 0.33, 95% CI 0.14–0.74, *p* = 0.008). Collectively, these findings offer some reference for individualized decision-making in the peri-procedural management of endovascular stroke therapy.

The central findings of this study—namely, the positive trend for Non-GA in the overall cohort coupled with a potential benefit in the mild-stroke subgroup—offer a novel perspective for reconciling longstanding controversies within this domain. The conclusion of the HERMES Collaboration meta-analysis, indicating that GA leads to an elevated rate of poor prognosis in patients with anterior circulation large-vessel occlusion stroke undergoing MT, aligns directionally with our observed trend (15). Post-hoc analyses from the ESCAPE-NA1 trial revealed that GA significantly increased the incidence of early neurological deterioration following endovascular therapy (EVT) (16), and an analysis of the MR CLEAN registry demonstrated significantly worse 90-day functional outcomes for patients receiving GA compared to those under local anesthesia (17). Conversely, the SIESTA, ANSTROKE, and GOLIATH trials each concluded that the choice of anesthetic technique did not significantly influence functional outcomes (18–20), though a subsequent meta-analysis of these three single-center randomized controlled trials (RCTs) paradoxically suggested that GA was associated with significantly improved 90-day functional outcomes (21). Recent multicenter RCTs have also yielded inconsistent results: the AMETIS trial found no significant difference in functional independence rates or major peri-procedural complications between GA and procedural sedation (22), while the SEGA trial reported superior functional outcomes and higher reperfusion success rates with GA (23). This heterogeneity largely stems from confounding bias and population heterogeneity inherent in different study designs. In observational analyses, the tendency to select patients with more severe strokes and poorer baseline prognoses for GA profoundly inflates its apparent detrimental effect (8). Our data vividly illustrate this confounding influence: the Non-GA group demonstrated a trend toward better clinical outcomes in unadjusted analyses, however, following rigorous adjustment for baseline imbalances—including stroke severity, comorbidities, and reperfusion times—the overall difference, though attenuated, persisted in favor of Non-GA. This residual positive trend may reflect inherent physiological advantages of a Non-GA strategy after accounting for major confounders, such as the potential for better preservation of cerebral autoregulation, avoidance of anesthetic agent-induced suppression of cerebral metabolism, and mitigation of the effects of positive-pressure ventilation on cerebral venous return and intracranial pressure (6, 24–27). Nevertheless, within a heterogeneous overall population, these potential benefits may be diluted by other, more potent determinants of final outcome—such as initial infarct core volume and the degree of collateral circulation—thereby precluding the attainment of conventional statistical significance for the primary endpoint (28–30).

The results of the subgroup analysis are particularly illuminating, revealing baseline stroke severity as a key modifier of anesthetic effect. Theoretically, for patients with an NIHSS score <15, who likely possess a relatively larger ischemic penumbra and greater neuroplastic potential (31–33), the maintenance of physiological homeostasis afforded by avoiding GA—including more stable autonomous blood pressure, preserved cough reflex and airway protective mechanisms, and the absence of residual anesthetic effects—may be especially critical for facilitating neurological recovery (34). By minimizing peri-procedural physiological perturbations, Non-GA may foster a more optimal microenvironment for brain tissue repair in this specific subgroup. Conversely, for patients with severe strokes (NIHSS score ≥15), the extent of initial cerebral injury is often profound, and functional outcomes may be largely predetermined by the index insult, thereby limiting the marginal impact of anesthetic choice. Furthermore, these critically ill patients frequently present with impaired consciousness, compromised airways, or severe agitation, situations in which the imperative to ensure procedural safety and feasibility makes GA a clinical necessity, potentially offsetting its theoretical physiological disadvantages (35). It should be emphasized that the above mechanistic interpretations are speculative, and this study lacks direct physiological data to support these interpretations; the relevant mechanisms warrant further investigation in future studies.

Another finding of this study is the association between GA and an increased risk of postoperative pneumonia, a result consistent with other clinical studies (18, 36, 37). This association has a sound pathophysiological basis: endotracheal intubation directly compromises the airway barrier, sedative agents impair mucociliary clearance and the cough reflex, and the combination of post-stroke dysphagia and immunosuppression may create conditions conducive to pulmonary infection (38, 39). Pneumonia is not only a common perioperative complication but also a well-established independent predictor of post-stroke mortality, delayed functional recovery, and increased healthcare costs (40–43). Therefore, this finding warrants consideration in clinical practice. The choice of anesthetic strategy may need to incorporate pulmonary risk into a comprehensive assessment; even for patients in whom general anesthesia is deemed necessary, our results suggest that attention to perioperative lung-protective measures—such as preoperative risk assessment, intraoperative lung-protective ventilation, prompt postoperative extubation, meticulous oral care, and systematic screening for swallowing dysfunction—may have clinical value.

The principal strength of this study lies in its foundation within a high-quality, multicenter randomized controlled trial, featuring prospectively collected data, and adjustment for a comprehensive array of potential confounding variables, thereby yielding evidence more robust than that derived from purely observational studies. Several limitations warrant acknowledgement. First, the non-randomized assignment of anesthetic modality, while addressed through multivariable adjustment, precludes the complete exclusion of residual confounding. Second, the “Non-GA” category amalgamates a spectrum from local anesthesia alone to varying depths of sedation, preventing a more granular analysis of these distinct approaches. Third, detailed data regarding the depth of GA, specific pharmacological agents employed, and precise intraoperative hemodynamic fluctuations were not available. In addition, no multiplicity adjustment was made for our secondary outcomes and subgroup analyses, as these were exploratory analyses. Another limitation is the failure to document patients who converted from non-general anesthesia to general anesthesia during the procedure, which may have introduced some bias into the results. Finally, the failure to clearly define the specific type of pneumonia and document it represents another limitation.

Building upon the insights from this investigation, future research efforts should concentrate on several key avenues. First, there is a need for dedicated RCTs specifically targeting patients with mild-to-moderate stroke (e.g., NIHSS <15) to compare optimized Non-GA protocols against standard GA, thereby validating the reliability of our subgroup finding. Second, deeper exploration into the optimal implementation of Non-GA is required, including defining the ideal sedation depth, identifying optimal drug regimens, and establishing criteria to prevent unplanned conversion to GA. Third, the development and validation of clinical decision-support tools that integrate multifaceted information—such as NIHSS score, advanced neuroimaging characteristics, airway assessment, and comorbidities—could facilitate truly individualized anesthetic selection. Finally, continued efforts to refine GA management protocols are essential, focusing on pharmacological choices (e.g., agents with potential neuroprotective or lung-protective properties), stricter hemodynamic target management, and enhanced postoperative recovery pathways to mitigate its associated risks.

## 5 Conclusion

This multicenter investigation suggests that in patients undergoing mechanical thrombectomy for acute ischemic stroke, Non-GA may be associated with better functional outcomes in patients presenting with milder neurological deficits. GA was associated with an increased risk of postoperative pneumonia. Acknowledging the inherent limitations of this analysis, future studies with larger, prospective cohorts are warranted to validate these findings and to refine the optimal sedation strategy, including the identification of the most suitable pharmacological agents, for anesthesia management during acute stroke intervention.

## Data Availability

The original contributions presented in the study are included in the article/Supplementary material, further inquiries can be directed to the corresponding authors.
