# Supplementary material for: Impact of anesthesia methods on functional outcomes in patients with acute ischemic stroke undergoing mechanical thrombectomy: insights from the ENCHANTED2/MT trial
Source: Front Neurol. 2026 Apr 23;17:1736293. doi: 10.3389/fneur.2026.1736293 (PMC13149095; doi:10.3389/fneur.2026.1736293)
Supplement: Supplementary file 1 [file Supplementary_file_1.docx]

****Supplementary File 1****
****List of Ethics Committees at Each Participating Hospital****

| **No.** | **Participating Hospital** | **Ethics Committee Name** |
| --- | --- | --- |
| 1 | Shanghai Changhai Hospital | Ethics Committee of Shanghai Changhai Hospital |
| 2 | Zhoukou Central Hospital | Ethics Committee of Zhoukou Central Hospital |
| 3 | Liaocheng People's Hospital Brain Hospital | Ethics Committee of Liaocheng People's Hospital Brain Hospital |
| 4 | The Second Affiliated Hospital of Bengbu Medical College | Ethics Committee of the Second Affiliated Hospital of Bengbu Medical College |
| 5 | Wenzhou Central Hospital | Ethics Committee of Wenzhou Central Hospital |
| 6 | Nanyang Central Hospital | Ethics Committee of Nanyang Central Hospital |
| 7 | Shenyang First People's Hospital | Ethics Committee of Shenyang First People's Hospital |
| 8 | Linyi People's Hospital | Ethics Committee of Linyi People's Hospital |
| 9 | Zhangzhou Municipal Hospital of Fujian Province | Ethics Committee of Zhangzhou Municipal Hospital of Fujian Province |
| 10 | Chongqing University Three Gorges Hospital | Ethics Committee of Chongqing University Three Gorges Hospital |
| 11 | Nanning Second People's Hospital | Ethics Committee of Nanning Second People's Hospital |
| 12 | Qingpu Branch of Zhongshan Hospital, Fudan University | Ethics Committee of Qingpu Branch of Zhongshan Hospital, Fudan University |
| 13 | Changzhou First People's Hospital | Ethics Committee of Changzhou First People's Hospital |
| 14 | The 904th Hospital of Joint Logistics Support Force of PLA | Ethics Committee of the 904th Hospital of Joint Logistics Support Force of PLA |
| 15 | Taizhou First People's Hospital | Ethics Committee of Taizhou First People's Hospital |
| 16 | Yunfu People's Hospital | Ethics Committee of Yunfu People's Hospital |
| 17 | The First Affiliated Hospital of Anhui Medical University | Ethics Committee of the First Affiliated Hospital of Anhui Medical University |
| 18 | Heze Municipal Hospital | Ethics Committee of Heze Municipal Hospital |
| 19 | Zhuhai People's Hospital | Ethics Committee of Zhuhai People's Hospital |
| 20 | Henan Provincial People's Hospital | Ethics Committee of Henan Provincial People's Hospital |
| 21 | Army Medical Center (Daping Hospital) | Ethics Committee of Army Medical Center (Daping Hospital) |
| 22 | Tianjin Huanhu Hospital | Ethics Committee of Tianjin Huanhu Hospital |
| 23 | PLA Strategic Support Force Medical Center | Ethics Committee of PLA Strategic Support Force Medical Center |
| 24 | Aerospace Center Hospital | Ethics Committee of Aerospace Center Hospital |
| 25 | Luoyang Central Hospital | Ethics Committee of Luoyang Central Hospital |
| 26 | Maoming Hospital of Traditional Chinese Medicine, Guangdong Province | Ethics Committee of Maoming Hospital of Traditional Chinese Medicine, Guangdong Province |
| 27 | Jiangsu Provincial People's Hospital | Ethics Committee of Jiangsu Provincial People's Hospital |
| 28 | Zhejiang Hospital | Ethics Committee of Zhejiang Hospital |
| 29 | Hangzhou First People's Hospital | Ethics Committee of Hangzhou First People's Hospital |
| 30 | Dalian Central Hospital | Ethics Committee of Dalian Central Hospital |
| 31 | The First Affiliated Hospital of University of Science and Technology of China | Ethics Committee of the First Affiliated Hospital of University of Science and Technology of China |
| 32 | The First Affiliated Hospital of Soochow University | Ethics Committee of the First Affiliated Hospital of Soochow University |
| 33 | Ningbo First Hospital | Ethics Committee of Ningbo First Hospital |
| 34 | Cangzhou Central Hospital | Ethics Committee of Cangzhou Central Hospital |
| 35 | China-Japan Union Hospital of Jilin University | Ethics Committee of China-Japan Union Hospital of Jilin University |
| 36 | Peking University Binghai Hospital (Tianjin Fifth Central Hospital) | Ethics Committee of Peking University Binhai Hospital (Tianjin Fifth Central Hospital) |
| 37 | The First Affiliated Hospital of Xi'an Jiaotong University | Ethics Committee of the First Affiliated Hospital of Xi'an Jiaotong University |
| 38 | The First Affiliated Hospital of Jinan University | Ethics Committee of the First Affiliated Hospital of Jinan University |
| 39 | Lishui Central Hospital | Ethics Committee of Lishui Central Hospital |
| 40 | Jinan Central Hospital | Ethics Committee of Jinan Central Hospital |
| 41 | Shanghai Tenth People's Hospital | Ethics Committee of Shanghai Tenth People's Hospital |
| 42 | Hunan Provincial People's Hospital | Ethics Committee of Hunan Provincial People's Hospital |
| 43 | Wuhan First Hospital | Ethics Committee of Wuhan First Hospital |
| 44 | Western Theater Command General Hospital of PLA | Ethics Committee of Western Theater Command General Hospital of PLA |
